# Efficacy and tolerability of sulforaphane in the therapeutic management of cancers: a systematic review of randomized controlled trials

**DOI:** 10.3389/fonc.2023.1251895

**Published:** 2023-11-24

**Authors:** Dana ElKhalifa, Nour Al-Ziftawi, Ahmed Awaisu, Feras Alali, Ashraf Khalil

**Affiliations:** ^1^ Department of Pharmacy, Aspetar Orthopedic and Sports Medicine Hospital, Doha, Qatar; ^2^ Department of Pharmacy, Aman Hospital, Doha, Qatar; ^3^ College of Pharmacy, QU Health, Qatar University, Doha, Qatar

**Keywords:** cancer, sulforaphane, isothiocyanates, glucoraphanin, broccoli, cruciferous vegetables

## Abstract

**Objectives:**

This paper presents a systematic review aimed at assessing the therapeutic potential of sulforaphane (SFN) in the treatment of diverse cancer types.

**Methods:**

Following Cochrane guidelines for systematic reviews, we conducted an exhaustive search of electronic databases up to May 12, 2023, encompassing PubMed, Cochrane, Embase, Web of Science, Google Scholar, Natural Medicines, ProQuest, ClinicalTrials.gov, and ICTRP. Studies were included if they were human-based RCTs involving cancer patients where SFN was the primary experimental treatment. The Cochrane Risk of Bias tool for RCTs (RoB2) was used for quality assessment.

**Results:**

Eight studies investigating the efficacy and safety of SFN in prostate cancer (PCa), breast cancer, pancreatic cancer, and melanoma were identified and included in the review. The dosing regimens were variable and inconsistent across the studies. SFN treatment led to statistically significant alterations in several vital genes and histological biomarkers across the studies. However, it did not impact some other key genes. Although not statistically significant, SFN improved overall survival in pancreatic cancer patients. The results on prostate-specific antigen (PSA) were inconsistent in PCa. None of the studies reported significant differences between SFN and comparative controls in terms of adverse events.

**Conclusion:**

SFN has emerged as a promising and safe therapeutic agent for diverse cancer types. Nevertheless, the high levels of methodological and clinical heterogeneity across the included studies precluded the possibility of conducting meta-analyses. Further robust clinical investigations to conclusively ascertain the chemotherapeutic potential of SFN in the management of various cancer forms are needed.

**Systematic review registration:**

https://www.crd.york.ac.uk/prospero/display_record.php?ID=CRD42022323788, identifier CRD42022323788.

## 1 Introduction

**Figure 1:**
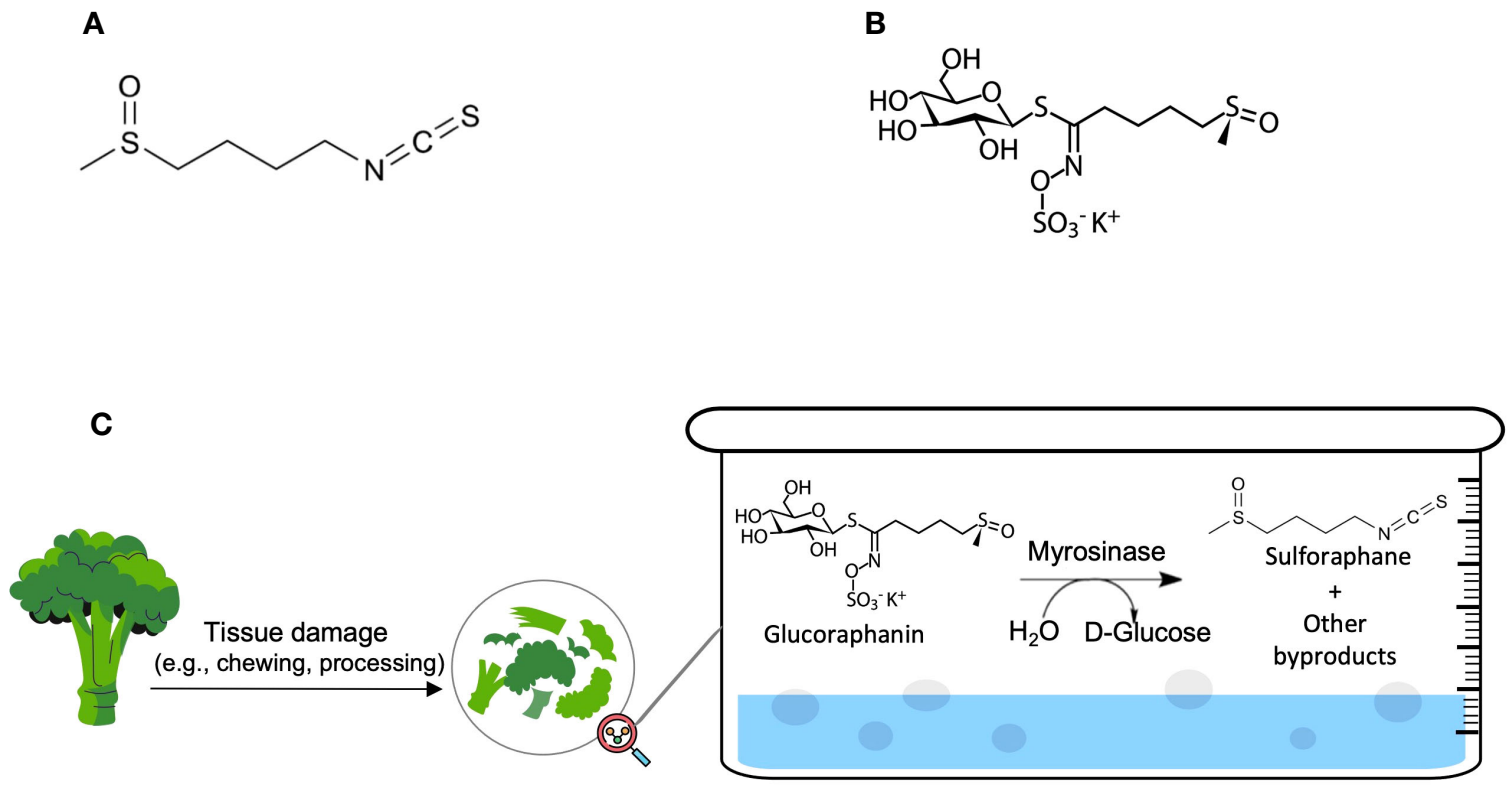
**(A)** Sulforaphane (C_6_H_11_NOS_2_) and **(B)** Glucoraphanin (C_12_H_23_NO_10_S_3_) molecular structures; **(C)** The natural chemical process of SFN production.

Cancer is one of the most significant public health problems and a leading cause of death worldwide (1). Several synthetic and natural chemotherapeutic agents have been discovered and used in the treatment of various types of cancers to increase overall survival and minimize symptoms (2). Natural active molecules were reported to constitute around 75% of the current anticancer medications (2). SFN is considered as an invaluable natural substance, richly available in plants, and having potential anticancer activity (3). Its chemical name is 1-Isothiocyanato-4-(methylsulfinyl)butane (chemical structure shown in **Figure 1A**). SFN is a sulfur-containing isothiocyanate that is mainly derived from the family of Brassicaceae flowering plants, also known as ‘Cruciferae’ (3). Among the Brassicaceae species is *Brassica oleracea*, which includes some widespread edible vegetables such as broccoli, cabbage, brussels sprouts, cauliflower, and kale (4). The most studied secondary metabolites in Brassicaceae vegetables are glucosinolates, which are water-soluble molecules containing a variable aglycone side chain and a common β-D-thioglucose moiety (4). They are Sulphur-rich, nitrogen-containing anionic products (4). SFN exists in particularly high amounts in broccoli, broccoli sprouts, and cabbage in the form of its glucosinolate precursor, glucoraphanin (4-methylsulphinylbutyl glucosinolate) (**Figure 1B**) (5–7).

The natural production of SFN occurs principally via a chemical reaction between glucoraphanin and plant thioglucosidases (‘myrosinases’) after tissue damage or, if myrosinases were denatured via cooking before freezing, the reaction will occur between glucoraphanin and microbial thioglucosidases in the colon (5–7). The hydrolysis products include goitrin, several isothiocyanates, thiocyanate ions, and nitriles. **Figure 1C** demonstrates the mechanism of the hydrolysis of glucoraphanin by myrosinase and the subsequent production of its biologically active metabolite, SFN. Amongst the hydrolysis products, isothiocyanates (e.g., SFN) were reported to possess protective and anticarcinogenic effects when consumed in a cruciferous-rich diet (5–7).

SFN has been reported to have therapeutic and medicinal effects in the treatment of cancer, autism, *Helicobacter pylori*, asthma, and liver disease (8–13). It was postulated to possess anticancer effects through different molecular mechanisms (14). These mechanisms include oxidants and carcinogens detoxification, apoptosis, G2/M and G1 cell cycle phase arrest, phase I and II metabolic enzymes blockade (e.g., cytochrome P450 2E1 [CYP2E1] and CYP1A2, and glutathione-S-transferase), angiogenesis and metastasis inhibition, downregulation of histone deacetylase activity, epigenetic modifications, and cell proliferation inhibition (15–18). It was also reported to embrace synergistic anticancer effects when combined with paclitaxel, gemcitabine, doxorubicin, 5-fluorouracil, or cisplatin, which may help in reducing their recommended doses (18, 19). SFN is considered safe when consumed orally in food and was shown to be safe in clinical studies when consumed in medicinal doses ranging between 9 and 60 mg in different types of disease conditions (20).

To date, the use of SFN for the treatment and prevention of different types of cancers is supported and widely investigated in preclinical, clinical, and epidemiological studies (16, 21, 22). It is unclear how significant are the added benefits of SFN in patients with a confirmed diagnosis of cancer and whether it should be recommended as an additional intervention in cancer treatment guidelines when supplied from dietary sources or as an extract. In addition, there are no systematic reviews to synthesize the available evidence of the safety and efficacy of SFN in the treatment of cancer. Therefore, we aimed to systematically review the available evidence of RCTs regarding the efficacy and tolerability of SFN as a monotherapy or adjuvant therapy in the management of different types of cancers.

## 2 Methods

### 2.1 Protocol and registration

The protocol of this systematic review was registered in the PROSPERO database (ID: *CRD42022323788*). No changes to the protocol were made while conducting this systematic review. The Cochrane Handbook for Systematic Reviews of Interventions guidelines were followed according to the Preferred Reporting Items for Systematic Reviews and Meta-Analyses (PRISMA) statement (23, 24).

### 2.2 Search strategy

A Comprehensive broad search was performed using the following bibliographic databases: PubMed, Cochrane Library, EMBASE, Web of Science, and Google Scholar. We also searched the Natural Medicines database, which includes data on the comparative effectiveness of different natural products, to identify any available clinical trials meeting our inclusion criteria. Furthermore, the references of eligible and potentially eligible records were manually screened for eligibility. In addition, ClinicalTrials.gov, ProQuest, and WHO International Clinical Trials Registry Platform (ICTRP) search portals were searched to identify gray literature. The search was conducted to identify studies published up to May 12, 2023.

The search was composed of two main domains combined using appropriate Boolean operators: cancer and sulforaphane. Filters included English language and human studies. The complete search strategy for each of the databases can be found in the **Supplementary Material**. The removal of duplicates, title/abstract screening, and full-text screening was conducted using the Rayyan application for systematic reviews (25). Titles and abstracts screening based on predefined eligibility criteria was performed by two independent reviewers (DK and NA) and any disagreements were resolved through consensus. Subsequently, full-text articles of potentially eligible studies were reviewed independently by the same reviewers for inclusion of eligible studies, and again any disagreements were resolved through consensus.

### 2.3 Eligibility and study selection

Records were included if they involve: i) patients with a confirmed diagnosis of cancer (any type and any stage); ii) patients from all age groups and genders; iii) RCTs; iv) SFN as an intervention regardless of formulation, dose, and duration. However, records were excluded if they involved: i) preclinical or non-randomized clinical studies; ii) the main intervention not focusing on SFN; iii) cancer preventive effect of SFN in healthy individuals; iv) articles not in English.

The characteristics of the included studies can be summarized using the population, intervention, comparison, and outcomes (PICO) model as follows:

- *P:* Patients with a confirmed diagnosis of any cancer type
- *I:* SFN as a primary or synergistic agent for cancer management.
- *C:* Placebo or any comparative treatment
- *O:* Overall survival, laboratory results of relevant biomarkers (e.g. PSA), histological and radiological results, gene expression results, and side effects.

### 2.4 Outcomes

The primary outcomes included all effectiveness indicators or biomarkers including the overall survival, disease progression biomarkers, laboratory results of relevant biomarkers (e.g. PSA), histological and radiological results, and gene expression results. Secondary outcomes included any reported adverse events.

### 2.5 Data extraction

A predesigned data extraction form was used for the extraction and summary of data from the studies. This data extraction sheet included the following information: authors, publication year, journal name and the journal quartile ranking, country, and setting of the study, study objectives, inclusion/exclusion criteria, sample size, patients’ characteristics, intervention and comparator description, sample size, duration, follow up, study outcomes, major findings, and study conclusion. The data extraction was independently performed by two authors (DK and NA). In case of missing or unclear information, the corresponding authors of the studies were contacted for clarification through email.

### 2.6 Risk of bias assessment

The risk of bias and methodological quality of the included RCTs was evaluated by two independent reviewers (DK and NA) using the RoB2 tool (26). RoB2 comprised five major domains: ‘randomization process’, ‘deviations from intended interventions’, ‘missing outcome data’, ‘measurement of outcomes’, and ‘selection of reported results. An overall rating of the risk of bias was obtained at the end. A bias value of “high risk,” “some concerns,” or “low risk” was given for each domain. According to the RoB2 guideline for risk of bias, a study was deemed to be of an overall ‘high risk of bias’ if any of the domains was scored as ‘high risk’; with a moderate risk of bias, or what is called ‘with some concerns’, if any of the domains was scored ‘some concerns’; and with ‘low risk’ of bias if all the domains were scored ‘low risk’. The algorithm was followed by the two assessors for the scoring of each study. Any disagreements between the authors were resolved by consensus. All eligible studies were included in the qualitative data synthesis regardless of their risk of bias.

### 2.7 Data synthesis and analysis

Studies were grouped based on the cancer type and synthesized based on their reported outcomes. Owing to the heterogeneity of the interventions, outcomes, and types of cancer that were identified in the studies, meta-analyses were not conducted.

## 3 Results

### 3.1 Studies selection

**Figure 2:**
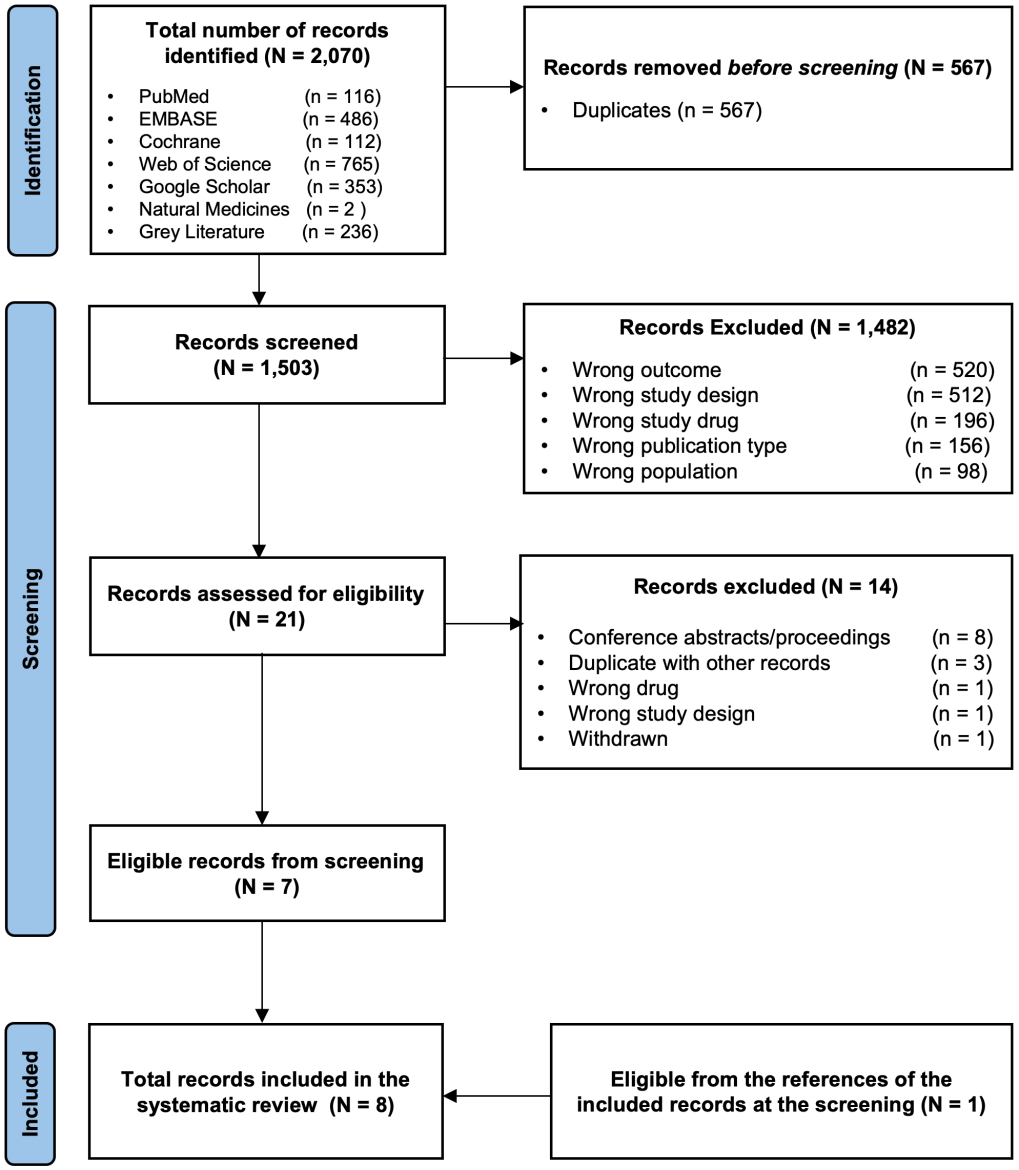
The PRISMA flow diagram for the systematic review records *From:* Page MJ, McKenzie JE, Bossuyt PM, Boutron I, Hoffmann TC, Mulrow CD, et al. The PRISMA 2020 statement: an updated guideline for reporting systematic reviews. BMJ 2021;372:n71. doi: 10.1136/bmj.n7.

A total of 2070 records were identified from the different databases. A total of 567 were removed because they were recognized as duplicates. Of the remaining 1503 records screened for titles and abstracts, 1482 were excluded. The remaining 21 potentially eligible records underwent full-text screening. Of these, 14 were excluded for the following reasons: conference abstracts/proceedings not meeting eligibility criteria (n = 8), duplicates that were not detected by the automation tools (n = 3), wrong intervention (n = 1), wrong study design (n = 1), and article withdrawn (n = 1). The remaining studies (n= 7) matched the inclusion criteria and were included in this systematic review. Further screening for the references of these seven included studies yielded one more eligible study, which was also included for the total studies analyzed in this review (n = 8). The detailed inclusion/exclusion process of the studies is summarized using the PRISMA flow diagram shown in **Figure 2**.

### 3.2 Studies characteristics

Detailed information on the publications is outlined in **Table 1**. Most of the included studies (n = 5) were published in high-quality journals, with Q1 ranking (9, 27, 28, 31, 32). The remaining articles were published in Q2 journals (n = 2) (8, 29) and ClinicalTrials.gov; (n = 1) (30). Although no time restrictions were applied, most of the included records (n = 7) were recent and published between 2015 and 2020 (8, 9, 27–31). Most of the included RCTs were conducted in the United States of America (USA) (n = 4) (8, 9, 29, 30).

**Table 1:** General information on the included publications.

Author(s)	Name of the Journal	Journal quartile ranking	Publication year	Country of study	Setting (location)
Zhang Z., et al. (8)	Nutrition and Cancer	Q2	2020	USA	Urology clinic at VA Portland Health Care System
Lozanovski V., et al. (27)	Investigational new drugs	Q1	2020	Germany	Department of General, Visceral & Transplant Surgery of the University of Heidelberg
Traka M., et al. (28)	The American Journal of Clinical Nutrition	Q1	2019	UK	Urology Department of the Norfolk and Norwich University Hospitals NHS Foundation Trust
Tahata S. et al. (29)	Cancer Prevention Research	Q2	2018	USA	UPMC Hillman Cancer Center
Visvanathan K., et al. (30)	Clinical Trials. Gov	N/A	2018	USA	Johns Hopkins Hospital
Atwell L., et al. (9)	Cancer Prevention Research	Q1	2016	USA	Oregon Health and Science University's (OHSU) Center for Women's Health Breast Center in Portland, OR
Cipolla B., et al. (31)	Cancer Prevention Research	Q1	2015	France	14 urological or oncological centers in France
Traka M., et al. (32)	PLoS ONE	Q1	2008	UK	Urology Department of the Norfolk and Norwich University Hospitals NHS Foundation Trust

The characteristics and outcomes of the included RCTs are summarized in **Table 2**, while the patients’ baseline characteristics are included in the **Supplementary Material** (**Table S9**). As presented in **Table 2**, PCa was the most commonly investigated cancer type (n = 4) (8, 28, 31, 32), followed by breast cancer (n = 2) (9, 30), whereas melanoma and pancreatic cancer were the least investigated cancer types (n = 1 for each) (27, 29). Based on our systematic literature search, no other cancers were studied in the RCTs investigating SFN as a therapeutic option. The reported outcomes varied greatly between the studies and within cancer types. They included different effectiveness and safety measures such as overall survival, relevant biomarkers, histological and gene expression results, and adverse events.

**Table 2:** Characteristics of included studies and their reported outcomes on the use of SFN in different cancer types.

Author & year	Aim of the study	No. of patients (intervention *vs*. control)	Intervention (dose, frequency, duration)	Comparator (dose, frequency, duration)	Type of cancer population	Primary outcome	Secondary outcome (if any)
Prostate cancer (PCa)							
Zhang Z., 2020 (8)	To determine the effects of short-term use of broccoli sprout extract on SFN metabolism and epigenetic biomarkers in PCa	n = 98 (50 *vs*. 49)	100 μmol of SFN BID x 4-8 weeks	Placebo (microcrystalline cellulose) x 4-8 weeks	PCa (benign/malignant)	1) Urinary, plasma, and prostate tissue SFN metabolites level; 2) HDAC activity; 3) IHC biomarkers; 4) prostate biopsy gene expression	N/A
Traka M., 2019 (28)	To evaluate whether glucoraphanin- rich broccoli soup use for 1 year can affect gene expression in PCa	n = 61 (Soup Y: 23; Soup Z: 18 *vs*. control: 20)	Broccoli Soup *Y:* 214 ± 7.3 μmol of glucoraphanin once weekly x 1 year AND Broccoli Soup *Z:* 492 ± 3.2 μmol of glucoraphanin once weekly x 1 year	Broccoli Soup X containing 72 ± 2.8 μmol 4-methylsulphinylbutyl glucosinolate (glucoraphanin) once weekly x 1 year	Low and intermediate risk PCa (early)	Gene expression in prostate tissue at baseline and after the dietary intervention	Changes in metabolites
Cipolla B., 2015 (31)	To investigate the effect of SFN at a daily dose of 60 mg for 6 months after radical prostatectomy	n = 78 (38 *vs*. 40)	60 mg of oral SFN (2 tablets containing 10-mg SFN each, 3 times a day) for 6 months, followed by 2 months without treatment	Placebo	Biochemically recurrent PCa after radical prostatectomy	The slope of log PSA determined from the values obtained between Month 0 and Month 6	Adverse events, PSA progression from baseline at month 6
Traka M., 2008 (32)	To compare changes in gene expression, PSA, and key polymorphic genes following a 12-month broccoli or peas in subjects with PCa	n = 21 (13 *vs*. 8)	400 g broccoli (containing 10.6 μmolesg^-1^ of 4-methylsulphinylbutyl and 3.6 μmolesg^-1^ of 3-methyl-sulphinylpropyl glucosinolates (per 100 g) per week for 12 months	400 g peas per week for 12 months	High-grade prostatic intraepithelial neoplasia	Changes in gene expression, PSA	NA
Breast cancer							
Visvanathan K., 2018 (30)	To examine the effect of a broccoli sprout on specific factors in breast cancer and assess whether SFN increases the levels of protective enzymes	n = 34 (17 *vs*. 17)	100 µmols of SFN (dissolved in 150 mL mango juice) once a day x 14 days	Placebo (150 mL mango juice without broccoli sprout ) OD x 14 days	Breast (DCIS)	Absolute change in mean proliferative rate measured by Ki67%	Phase II protein expression as assessed by changes in cytoprotective enzyme expression within tumor
Atwell L., 2015 (9)	To evaluate the efficacy of a broccoli sprout extract in altering HDAC activity and improving biomarkers for prognosis in benign or DCIS breast cancer	n = 54 (27 *vs*. 27)	BroccoMax™ containing glucoraphanin: 2 pills 3 times/day (TDD: 224 mg) for 2-8 weeks	Placebo	Women with abnormal mammograms: benign, DCIS, or IDC	1) Plasma and urinary SFN metabolites; 2) PBMC, HDAC activity, and tissue biomarkers (H3K18ac, H3K9ac, HDAC3, HDAC6, Ki-67, p21)	NA
Melanoma							
Tahata S., 2018 (29)	To evaluate the toxicity and potential effects of oral BSE-SFN at different dosages in melanoma	n = 17 (50, 100, and 200 μmol: 6 *vs*. 6 *vs*. 5)	100 or 200 μmol of oral BSE-SFN once daily for 28 days	50 μmol of oral BSE-SFN once daily for 28 days	Melanoma (at least 2 atypical nevi or a prior history of melanoma)	1) SFN concentration in plasma and skin; 2) gross and histologic changes in atypical nevi; 3) IHC biomarkers; 4) plasma cytokine levels; 5) protein expression	N/A
Pancreatic cancer							
Lozanovski V., 2020 (27)	To evaluate the feasibility of SFN-rich broccoli sprouts in patients with advanced PDA undergoing palliative chemotherapy	n = 40 (29 *vs*. 11)	Broccoli sprout (6 mg SFN per capsule): dosed at 15 capsules/day to reach a total of 90 mg plus approximately 180 mg glucoraphanin per day for 1 year	Placebo (methyl-cellulose)	Advanced pancreatic cancer	Overall survival rate at 3, 6, 9 and 12 months after the trial	NA

No., Number; vs, versus; N/A, Not applicable; SFN, sulforaphane; BID, twice a day; PCa, prostate cancer; HDAC, Histone deacetylases; IHC, immunohistochemistry; PSA, prostate specific antigen; OD, once daily; DCIS, Ductal carcinoma *in situ*; TDD, total daily dose; IDC, invasive ductal carcinoma; PBMC, peripheral blood mononuclear cell; BSE-SFN, broccoli sprout extract containing sulforaphane; PDA, Pancreatic ductal adenocarcinoma.

### 3.3 Risk of bias of included studies

**Figure 3:**
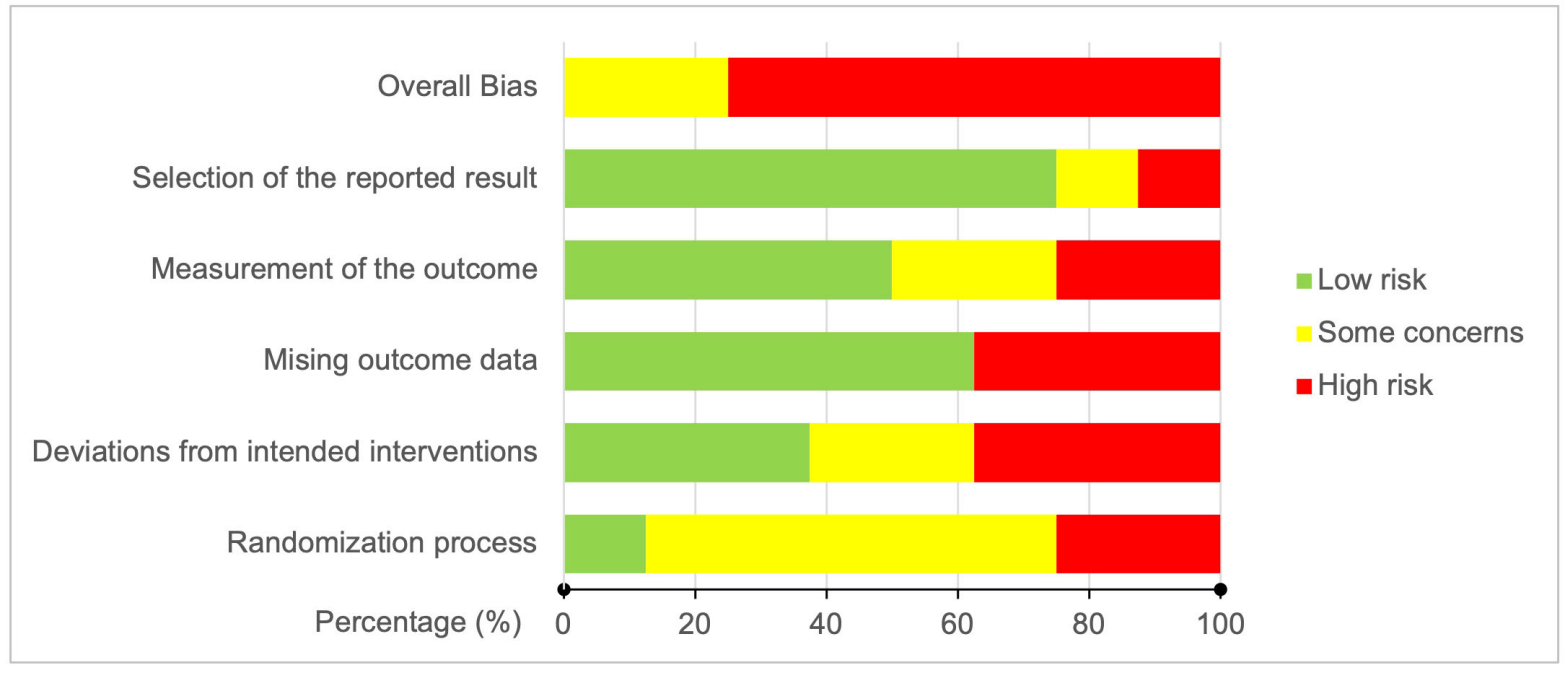
Risk of bias graph: Bias judgment percentages on each risk of bias domain across all included studies.

**Figure 4:**
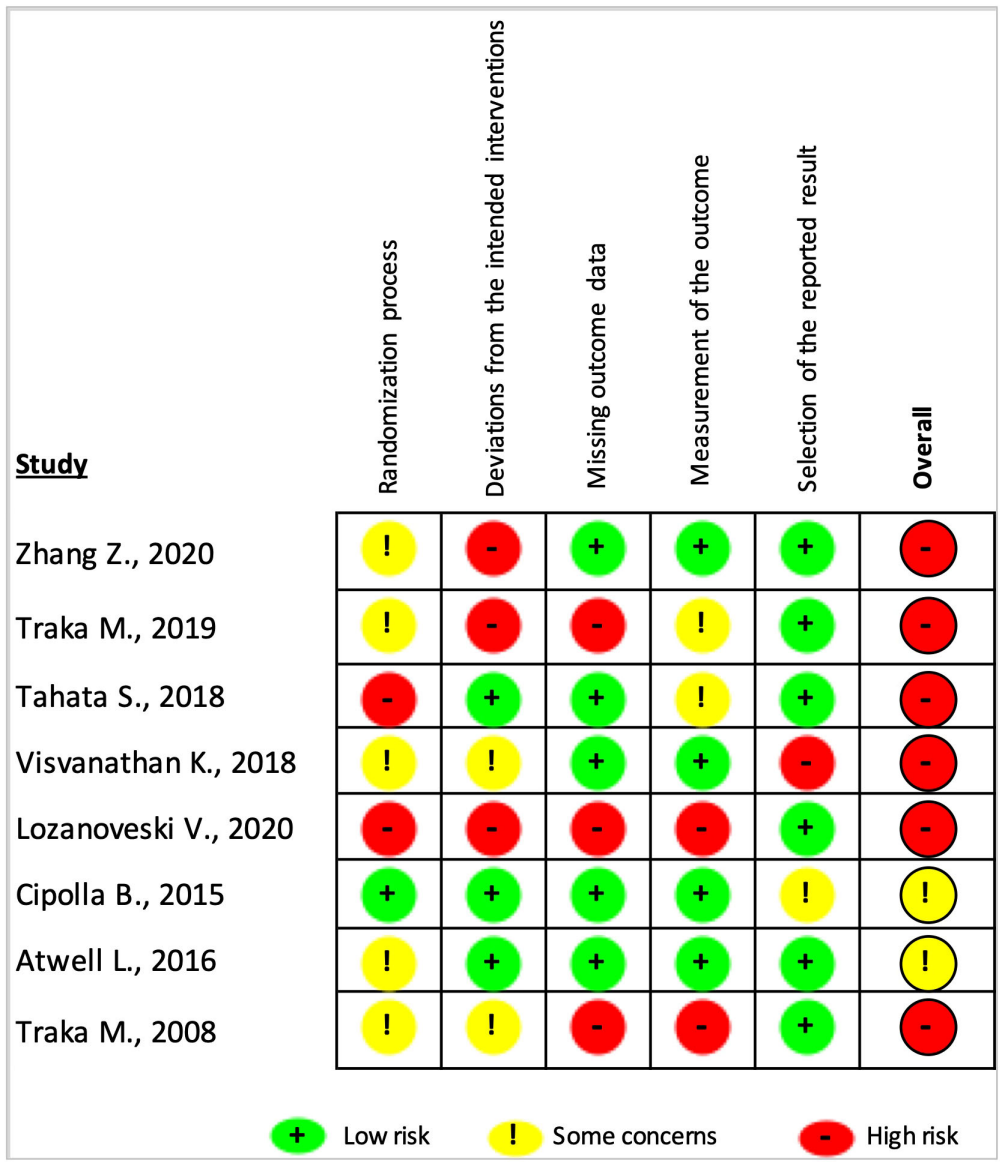
Risk of bias judgments summary for each risk of bias domain per individual study. The overall risk of bias was considered ‘high risk’ or ‘some concern’ if the study had at least one ‘high risk’ or ‘some concern’ rating in any of the five domains.

The results of the risk of bias assessment of the included articles using the RoB-2 tool are presented in **Figures 3**, **4**. Overall, there was a 75% high risk of bias in all the included clinical trials (**Figure 3**). Only two of the studies were reported to have ‘some concern’ (9, 31), while the remaining six studies were assessed as ‘high risk of bias’ (8, 27–30, 32). Particularly, the high risk occurred due to ‘missing outcome data’ and ‘deviations from intended interventions’ (37.5% each), followed equally by ‘randomization process’ and ‘measurement of the outcomes’ (25% each). As shown in **Figure 4** for individual studies, only one study had a low risk of bias in the ‘randomization process’ (31).

### 3.4 Data synthesis

Based on our systematic literature review findings, the effects of SFN were explored only in PCa, breast cancer, melanoma, and pancreatic cancer. The efficacy outcomes in the trials included overall survival, lab results of relevant biomarkers, histological findings, and gene expression findings. The safety outcomes included any reported adverse events. The dosing regimens and dosage forms of SFN were variable across the studies and are discussed below before the results on individual outcomes.

#### 3.4.1 SFN composition and preparations

**Figure 5:**
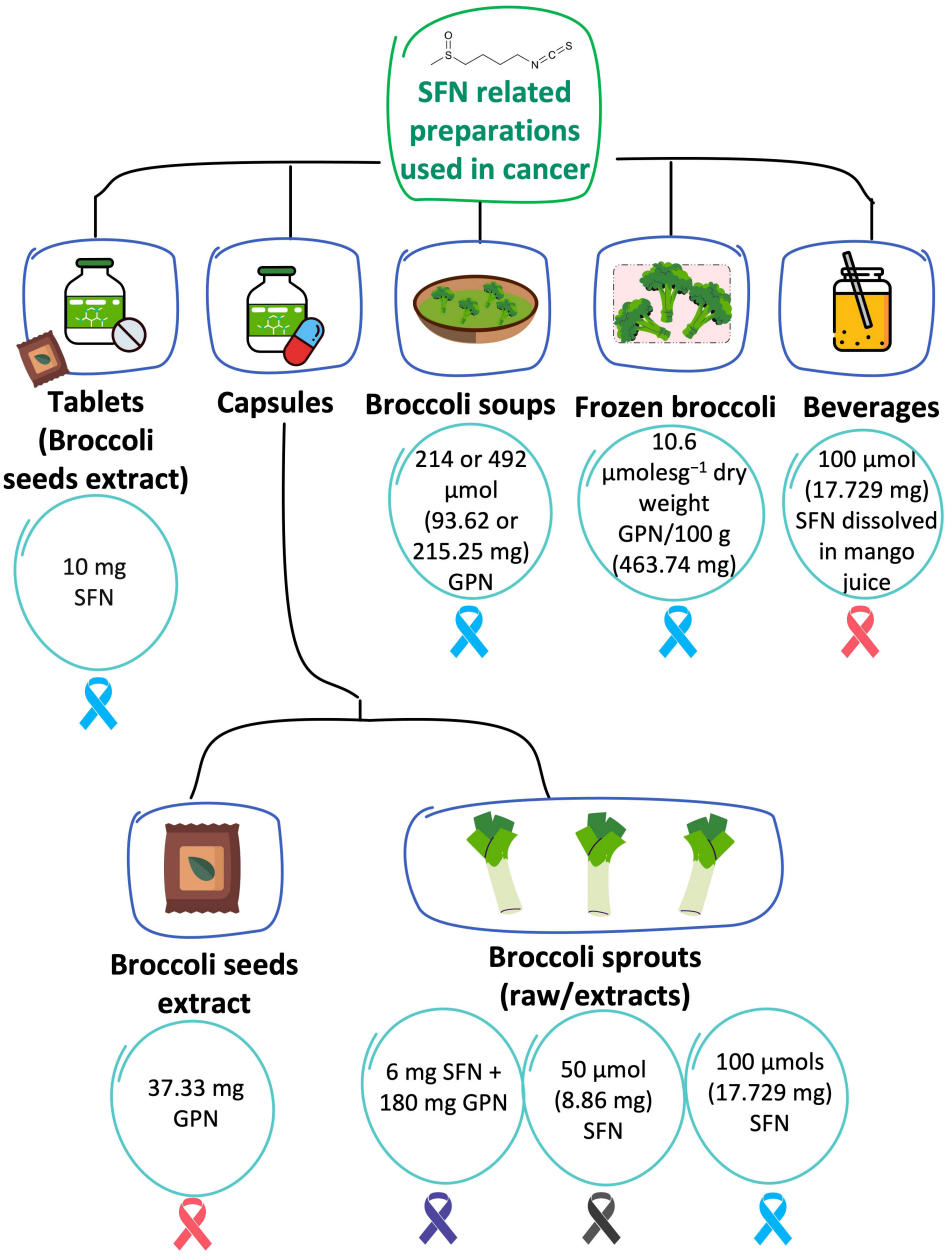
SFN-based preparations that were tested in cancer. The content per one portion of each preparation (in each of the 8 RCTs) is described in the circles. The investigated type of cancer is illustrated in ribbons color coded based on their related cancers: Blue is PCa; pink is breast cancer; purple is pancreatic cancer; black is melanoma. GPN refers to glucoraphanin. When the units of SFN/GPN were reported in μmol, they were converted to mg using SFN molecular weight of 177.29 g/mol or GPN molecular weight of 437.493 g/mol.

Different formulations of SFN were used in the included trials, which were all exclusively administered via the oral route. The investigated duration of therapy for the different interventions was highly varied and ranged from 2 weeks (30), 2-8 weeks (9), 28 days (29), 4-8 weeks (8), 6 months (31), to 12 months (27, 28, 32). Additionally, the preparation techniques and sources of the SFN were variable and included purchasing ready-made commercial products, formulating new products, and using frozen vegetables and extracts. Five types of formulations or preparations were used in the eight trials, including capsules (n = 4) (8, 9, 27, 29), tablets (n = 1) (31), broccoli soups (n = 1) (28), frozen broccoli (n = 1) (32), and SFN dissolved in juice (n = 1) (30). **Figure 5** summarizes these preparations, the content per portion, and the type of cancer they were investigated in. The exact regimes, including, the dose, frequency, and duration of therapy are further elaborated in the subsequent sections.

##### 3.4.1.1 Capsules

Capsules were the most used formulations and were used in four of the included trials (8, 9, 27, 29). All of the studies administered broccoli sprout extracts, except for one that has used broccoli seed extract (9). Atwell et al., randomized women with ductal carcinoma *in situ* (DCIS), or invasive ductal carcinoma (IDC) to consume 250 mg of a broccoli seed extract containing around 37.33 mg of glucoraphanin per capsule (~30 mg as reported by the manufacturer (BroccoMax) or placebo (9). The glucoraphanin content in BroccoMax was analyzed and no significant batch-to-batch variations were detected (9). The regimen was two pills three times per day (total daily dose of glucoraphanin: 224 mg as per content analysis) for 2-4 weeks (9). Post-intervention changes in total urinary and plasma SFN isothiocyanates and SFN metabolites (SFN-Cys, SFN-NAC, SFN and SFN-GSH in urine and SFN-NAC, SFN-GSH, and SFN-CG in plasma) were statistically significant in the SFN arm compared to the placebo (*P* <0.05) (9). In the study by Lozanovski et al., among patients with pancreatic cancer, SFN was supplied in the form of freeze-dried broccoli sprouts packaged in capsules, Dieters Broccoraphan^®^ (27). Each capsule contained 6 mg (34 μmol) SFN administered in a daily dose of 15 capsules to a total of 90 mg (508 μmol) SFN, in addition to approximately 180 mg (411 μmol) glucoraphanin per day for 1 year (27). In another study among patients with melanoma, an oral formulation of broccoli sprout extract containing sulforaphane (BSE-SFN) extracted from *Brassica oleracea* was used and the content of isothiocyanates and glucosinolates was analyzed and validated (29). Each capsule contained 218 mg of powder (containing 50 μmol SFN) (29, 33). The patients were randomized to receive one of three concentrations: 50, 100, or 200 μmol once daily for 28 days (29). Lastly, Zhang et al. investigated the effect of broccoli sprout extract (BSE) containing 100 μmol SFN per capsule twice a day for 4-8 weeks among patients with benign/malignant PCa (8).

##### 3.4.1.2 Tablets

Tablets were used in only one study (31). Cipolla et al. administered 10 mg tablets of free stabilized SFN extracted from broccoli seeds (prostaphane) to male patients with biochemical recurrence after radical prostatectomy (31). To improve SFN stability, immediate-release tablets of microencapsulated active component powder extract were developed and used (31). SFN tablets were administered in a daily dose of 60 mg (2 tablets containing 10 mg SFN each, 3 times a day) for 6 months (31). Patients were instructed not to change their usual dietary lifestyle (31).

##### 3.4.1.3 Broccoli soups

Broccoli soup was investigated in PCa (28). The patients were randomized to receive one of three broccoli soups administered in 300 mL portions once weekly for one year (28): Broccoli Soup Y containing 214 ± 7.3 μmol glucoraphanin, Broccoli Soup Z containing 492 ± 3.2 μmol glucoraphanin, or control of Broccoli Soup X containing 72 ± 2.8 μmol glucoraphanin (28). The once-weekly soup X was selected as the control because it was manufactured by a commercial cultivar of broccoli and was expected to be a part of a normal diet and not expected to play a significant role in cancer progression due to the low threshold of glucoraphanin concentration (28).

##### 3.4.1.4 Frozen broccoli

Traka et al. used 400 g of frozen broccoli (contains 10.6 μmolesg-1 of 4-methylsulphinylbutyl and 3.6 μmolesg-1 of 3-methyl-sulphinyl-propyl glucosinolates per 100 g) per week for 12 months among patients with high-grade prostatic intraepithelial neoplasia (32). To ensure consistency in glucosinolate content, the broccoli was grown in one batch at an experimental farm in the UK and was processed by a distinct company (32). Frozen portions at −18°C were delivered to the participants on a monthly basis and subjects were asked to steam them for 4–5 minutes (32). The levels, mean (SD), of 3-methyl-sulphinyl-propyl and 4-methylsulphinylbutyl glucosinolates (the precursors of iberin and SFN, respectively) were 3.6 (0.14) and 10.6 (0.38) µmolesg^−1^ dry weight, respectively, compared to 0.6 (0.01) and 4.4 (0.12) µmolesg^−1^ dry weight in broccoli purchased from local retail stores (32).

##### 3.4.1.5 Beverages

The study by Visvanathan et al. tested the effect of SFN dissolved in juice in the therapeutic management of DCIS. The investigators administered 100 µmols of SFN (dissolved in 150 mL of mango juice) once a day for 14 days for patients with DCIS (30).

#### 3.4.2 Efficacy outcomes

**Figure 6:**
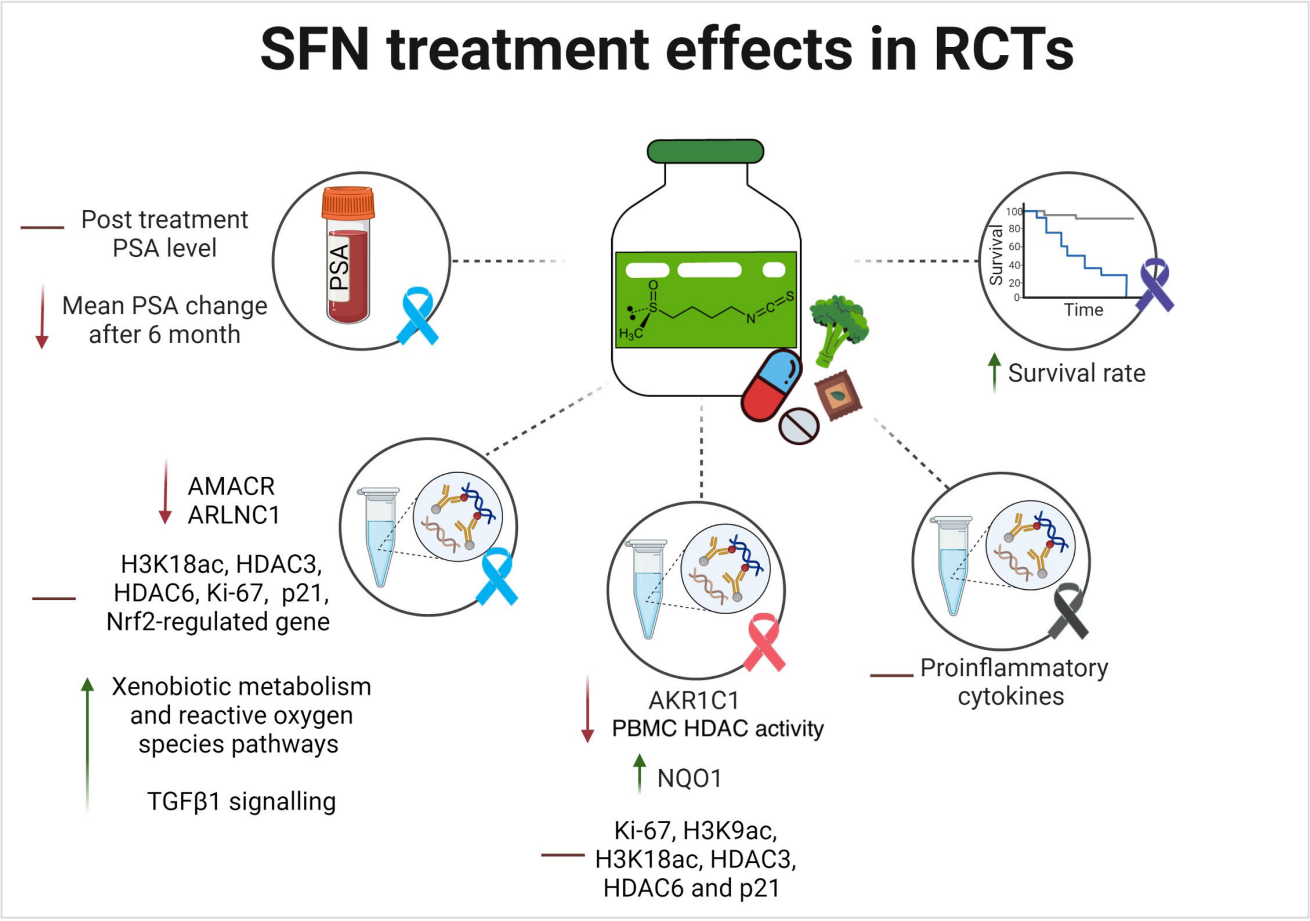
SFN reported efficacy outcomes in randomized human clinical trials. The lines indicates the following: ↑ indicates an increase/enhancement; ↓ indicates a decrease/reduction; ━ indicates no change. The investigated type of cancer is illustrated in ribbons color coded based on their related cancers: Blue is PCa; pink is breast cancer; purple is pancreatic cancer; black is melanoma.

Efficacy outcomes were determined using different surrogates and clinical endpoints as elaborated below. **Figure 6** illustrates the main efficacy findings from the included RCTs, which are summarized in the subsequent text.

##### 3.4.2.1 Effect on overall survival

Only one RCT investigated the overall survival rate in pancreatic cancer patients following treatment with SFN for 3, 6, 9, and 12 months (27). The study reported that the intake of 90 mg SFN in addition to 180 mg glucoraphanin daily for 6 months as compared to the placebo in advanced pancreatic cancer patients receiving palliative chemotherapy led to a lower mean death rate at 30, 90, and 180 days (day 30: 0% *vs*. 18%, day 90: 0% *vs*. 25%, and day 180: 25% *vs*. 43%) (27). A higher survival rate was also reported via Kaplan-Meier analysis (27). However, these findings were not statistically significant (*P=* 0.291 at day 180) (27). Additionally, there was a higher drop-out rate after 1 year (72% in the treatment group and 55% in the placebo group) (27).

##### 3.4.2.2 Effect on relevant tumor biomarkers

The main biomarker investigated for the possible anticancer effect of SFN was the change in PSA levels in PCa patients. Zhang et al., reported no PSA difference following SFN treatment for 4-8 weeks when compared to placebo in PCa and noncancer patients (8). In another study, no consistent changes in the levels of PSA levels were reported after 6 or 12 months in patients who received an SFN-rich diet as compared to the control (32). The third study that investigated the role of oral SFN in patients with biochemically recurrent PCa after radical prostatectomy reported that the mean changes in PSA levels between month 6 and baseline were significantly lesser in the SFN group than in the placebo group (+0.099 ± 0.341 ng/mL *vs*. +0.620 ± 1.417 ng/mL; *P=* 0.0433) (31). The PSA doubling time was also 86% lengthier in the SFN than in the placebo group (28.9 *vs*. 15.5 months, respectively). Following treatment, PSA slopes from 6 months to 8 months persisted at the same levels in the two arms (31).

##### 3.4.2.3 Effect of SFN on histological and gene expression findings

Several results on the expression of important genes and immunohistochemical markers were obtained from the included studies on three cancer types: PCa, breast cancer, and melanoma (8, 9, 28–30, 32). No data on pancreatic cancer were obtained.

In PCa, the main investigations were carried out on the expression of important proteins involved in PCa etiology, including AMACR, ARLNC1, epigenetic histone modifications, histone deacetylases (HDAC), Ki67 nuclear antigen, cyclin-dependent kinase inhibitors, nuclear factor-erythroid factor 2-related factor 2 (Nrf2), epidermal growth factor (EGF) (32), and transforming growth factor beta-1 (TGFβ-1) (8, 28, 32). The role of vital carcinogenesis processes and relevant signaling pathways, including epithelial-mesenchymal transition (EMT), inflammation, angiogenesis, apoptosis, xenobiotic metabolism, reactive oxygen, and insulin signaling were also explored by some of the trials (28, 32).

In total, 40 differentially expressed genes correlated with SFN treatment were identified in one study, including the downregulation of two genes previously implicated in PCa development (i.e. AMACR and ARLNC1) (8). A 4.3-fold lower level of the ARLNC1 gene was found among samples from PCa patients treated with an SFN-rich diet as compared to placebo, with a significant interaction between PCa and the effect of SFN intervention (*P=* 0.0281) (8). AMACR mRNA levels were seven-fold lower in PCa patients who took the intervention compared to placebo (*P* <0.0001) (8). No statistically significant differences were reported between SFN and placebo groups for all the examined tissue biomarkers (H3K18ac, HDAC3, HDAC6, Ki-67, and p21) in PCa and noncancer subgroups in the same study (8).

Furthermore, low-risk and intermediate-risk PCa (early) patients were recruited in a study investigating the anticancer role of three different concentrations of a broccoli soup containing glucoraphanin (214 μmol, 292 μmol in the treatment arms and 72 μmol in the control) once daily for 1 year (28). There were several hundred reported changes in gene expression in non-neoplastic tissue with enhanced expression of oncogenic genes (EMT and inflammation processes) in the control arm (*P* < 0.05) (28). Remarkably, those associated with angiogenesis (*P* < 0.001), apoptosis (*P* < 0.002), and androgen response (*P* < 0.001) were significantly enriched (28). In contrast, glucoraphanin-rich broccoli arms showed an inverse association between the consumption of the glucoraphanin-rich soup for 12 months and cancer progression (28). The intermediate dose of 214 μmol in soup Y showed highly a similar response to that of the control arm, soup X (28). Conversely, the high dose, soup Z (292 μmol of glucoraphanin), contrasted prominently with the control (soup X) in no augmentation of inflammatory and EMT responses (28). Besides, contrary to the control, there was a significant enhancement in downregulated genes for xenobiotic metabolism and reactive oxygen species pathways by soup Z (28). However, the study did not report any difference in the expression of any Nrf2-regulated gene at the beginning and end of the dietary intervention for all three treatment groups (*P* <0.1) (28). A third study determined the potential effects of broccoli rich in glucosinolates consumption on changes in gene expression in men with high-grade prostatic intraepithelial neoplasia (32). It involved a comparison between patients with positive and null GSTM1 allele (32). Following six months, significant changes between GSTM1 genotypes (positive and null) on the broccoli-rich diet associated with EGF and TGFb1 signaling pathways were noted (32). Those changes were pre-eminently present in the treatment arm than in the control pea-rich diet. Patients in the treatment group had additional changes in TGFb1 (*P=*  0.001), EGF (*P=*  0.068), and insulin signaling (*P=*  0.035) (32).

As for breast cancer, the role of SFN was mainly investigated on phase II proteins of cytoprotective enzymes, epigenetic histone modifications, HDAC, Ki67 nuclear antigen, and cyclin-dependent kinase inhibitors (9, 30). Visvanathan et al. explored the expression of phase II proteins of cytoprotective enzymes known to be altered in DCIS (AKR1C1 and NQO1) (30). At day 14 post-intervention, the mean of the percentage change (SD) of AKR1C1 expression in tumor cells was 62.1 (312.99) and 867.4 (2097.8) for SFN and placebo groups, respectively (30). For NQO1, the mean change after the treatment was 730.98 (2411.96) *vs*. 6.34 (30.12) for the SFN and the placebo groups, respectively (30). Conversely, in another study involving chemotherapy-naive patients with malignant breast tumors, there was no statistical significance between the groups [(Ki-67, H3K9ac, H3K18ac, HDAC3, HDAC6 and p21 levels in all the three examined tissue types (benign, DCIS or IDC)] in patients who received 224 mg of BroccoMax™ daily for 2-4 weeks when compared to those who received placebo (9). Nevertheless, within the treatment group, there was a significant reduction in Ki-67 (*P=* 0.003) and HDAC3 (*P=* 0.044) levels in benign tissue (9). The absolute change in mean proliferative rate, measured by Ki67%, was also investigated by Visvanathan et al. (30). In patients with breast cancer (DCIS), the mean change in Ki67% from baseline to 14 days post-intervention was -1.15 (2.08) in the treatment group versus 4 (17.08) in the placebo (30). Although there was a positive reduction in Ki67% with SFN, this effect was not statistically significant (*P=* 0.32) (30). In the study by Atwell et al., which investigated the change in HDAC activity pre- and post-intervention, there was -80.39 pmol/min/mg protein (p = 0.11) in the treatment group as compared to +27.52 pmol/min/mg protein (p = 0.40) in the placebo group (9). These changes in PBMC HDAC activity were significantly different (*P=* 0.04) between the two groups. The subgroup analysis stratified by NSAIDs use showed that among non-NSAID users, this change was particularly statistically significant (*P=* 0.04); while among NSAID users, the change was not significant (*P=* 0.30) (9).

In melanoma, only one study was conducted to investigate the effect of SFN treatment on proinflammatory cytokines (29). In patients with at least two atypical nevi or a prior history of melanoma, there was no significant correlation between the administration of three different concentrations (50, 100, and 200 μmol of oral SFN-rich diet once daily for 28 days) and changes in proinflammatory cytokines (29). However, when the data were pooled from all dosage groups, a statistically significant reduction in cytokines [MCP-1 (CCL-2), IP-10 (CXCL10), MIG (CXCL9), and MIP-1β (CCL-4)] were noted between days 1 and 28. However, alterations in IHC staining were not detected between days 1 and 28 (29).

#### 3.4.3 Safety outcomes

Concerning the safety outcomes, although few, the main reported side effects were gastrointestinal, including nausea, vomiting, diarrhea, bloating, flatulence, and constipation. Other documented side effects included taste alteration and headache. Only one patient across all studies was reported to drop out in the SFN group due to side effects (bowel discomfort) (31). In those who received 90 mg SFN in addition to 180 mg glucoraphanin daily for 6 months, Lozanovski V. et. al. reported increased digestive problems, flatulence, nausea, and vomiting, particularly in the treatment group (number of events and statistical significance were not reported) (27). In patients receiving 224 mg of BroccoMax™ daily for 2-4 weeks, few incidents of adverse events (including flatulence, bloating, nausea, vomiting, taste alteration, and headache) were reported in both groups (9). However, those were not statistically different and occurred in 8 (29.6%) patients in the treatment group and 9 (33.3%) patients in the control group (9). Additionally, the compliance rate was equivalent between the groups (*P=* 0.88) (9).

Mortality and serious adverse events were not reported at 14 days after 100 µmols of SFN dissolved in 150 mL mango juice (30). However, gastrointestinal side effects (constipation, nausea/vomiting, flatulence, diarrhea, and taste alteration) occurred in 9/15 (60%) patients in both groups (21 incidents in SFN and 17 in the placebo) (30). Similarly, toxicities and side effects were not reported after treatment with three different concentrations of BSE-SFN, except for grade 2 nausea that occurred in one patient in the 200 μmol group (29). In PCa (benign or malignant) patients treated with broccoli sprout extract containing 100 µmol of SFN twice a day for 4-8 weeks, no significant differences were noted between the two groups for adverse events (8). Only one adverse event of bloating and one adverse event of headache were reported in the treatment group and one taste alteration event in the placebo group (8). The compliance rate was high and similar in both groups (84% and 85%, respectively; *P=* 0.44) (8). Side effects were not investigated in the two studies by Traka and her colleagues (28, 32).

Adherence and safety were reported to be very good following consumption of 60 mg of oral SFN daily for 6 months (31). The difference in reported symptoms following treatment was not statistically significant as compared to placebo (*P=* 0.14) (31). Among all patients enrolled, 36 (44.4%) acknowledged at least one adverse event during the study (52.5% in the SFN arm and 36.5% in the placebo arm) (31). The adverse events were mainly (89%) grade 1 or at maximum grade 2 (11%). Grade 3/4 adverse events were not reported (31). Gastrointestinal adverse events (particularly bloating) were slightly greater in the SFN arm compared to the placebo arm (17 *vs*. 10). Only one patient in the SFN group withdrew after 1 month owing to bowel discomfort (31). Most of those side effects were short-term and were only reported once during the trial (31).

## 4 Discussion

This systematic review aimed at evaluating the available literature on the potential use of SFN as a therapeutic option for patients diagnosed with cancer. Eight RCTs were identified that investigated the potential anticancer effects of SFN in four types of cancer. The trials used various dosage formulations and dosing regimens of SFN and its glucosinolate precursor, glucoraphanin. Meta-analyses were not conducted due to the high level of methodological and clinical heterogeneity across studies including the different types of cancers that were identified. In addition, the GRADE (Grading of Recommendations, Assessment, Development, and Evaluations) approach was not for rating the certainty of evidence was not applied due to the limited number of reported results and patients identified per outcome. Therefore, we relied mainly on evaluating the risk of bias for all included studies as indicated in the protocol.

Despite the positive results, different pharmaceutical and dietary formulations and regimens were used, which made it challenging to determine the regimen that would give the desirable therapeutic outcomes. Among the eight trials, only few studies reported the bioavailability and absorption following the consumption of their SFN formulations (8, 9, 29). How significant the absorption and bioavailability were at different dosages and formulations are yet to be determined. In a pharmacokinetic study, myrosinase-treated broccoli sprout extract (BSE) was used at a dose of 200 μmol SFN once daily or at 100 μmol SFN twice daily in healthy adults (34). The study reported a high absorption and bioavailability of SFN following consumption, which resulted in high levels of SFN metabolites in urine and plasma (34). Particularly, the twice daily dosing was reported to retain greater plasma SFN metabolites compared to the 24-hour dosing (34). This may indicate that the various SFN formulations and inconsistent dosing frequencies and regimens might have impacted SFN absorption and efficacy in the different trials. In another pharmacokinetics cross-over trial, SFN-based beverages retained substantially greater SFN and SFN metabolites in urine following 12 hours of consumption as compared to GPN (35). However, the elimination rate was slower and steady with glucoraphanin (35). Based on this, preparations with combined SFN and its precursor, glucoraphanin, may retain more benefits. In a pilot study, the administration of the proton pump inhibitor, omeprazole, improved the conversion of glucoraphanin to SFN, possibly by sparing myrosinase enzyme from the acidic environment of the stomach (36). Therefore, enteric-coated formulations may be worthwhile for future considerations. Notably, before moving into large-scale RCTs, the best formulation, dose, route, and frequency have to be determined.

Efficacy endpoints were reached mainly when the interventions were supplied for longer durations of therapy. Shorter durations of a few weeks or a month rarely led to significant efficacy outcomes. The main explored efficacy endpoints in the trials included overall survival, relevant tumor biomarkers, and histological and gene expression results. Although some significant results were obtained in the studies, only one focused on overall survival, which has frequently been considered the ‘gold standard’ tangible treatment endpoint (27). The study supported the role of SFN in improving survival rate, but there was a high dropout, resulting in non-significant findings (27). Remarkably, the study had methodological limitations and a high level of bias as indicated in the risk of bias assessment. Furthermore, other important oncological clinical endpoints were not considered in any of the studies, including progression-free survival, response rate, complete response, and pathological complete response.

Since PSA was found to be highly implicated in PCa pathogenesis, it is considered as a vital biomarker for PCa screening and determination of treatment response (37, 38). Consequently, it was the main laboratory-related outcome investigated for the possible anticancer effect of SFN in PCa patients (8, 31, 32). The studies did not report a significant difference in PSA levels between baseline and post-treatment (8, 32). However, Cipolla et al., reported that the mean change in PSA levels was significantly lower in the SFN group compared to the placebo at 6 months and that the rate of PSA increase was also significantly lower in the treatment arm (31). Likewise, these studies were found to possess moderate to high risks of bias, and some had determined the response after a very short time period (i.e., 4 weeks). Given that PSA is not always PCa-specific and can be attenuated by other factors, SFN response on PSA should take into consideration other confounding factors when analyzing treatment response, which was lacking in the current trials.

Furthermore, most of the studies focused on immunohistochemical and gene expression outcomes. In PCa, SFN was reported to significantly attenuate ARLNC1 and AMACR genes (8). Both genes were reported to be involved in PCa progression, which would support the potential role of SFN in PCa treatment (37, 38). Similarly, in patients with a positive GSTM1 genotype, SFN led to significant changes in the EGF, TGFb1, and insulin signaling pathways (32). As described in the literature, those with GSTM1 allele deficiency are at increased susceptibility to developing PCa (39–41). This would postulate SFN potential as a promising chemoprevention modality in this cancer type. SFN has also positively attenuated EMT response, xenobiotic metabolism, and reactive oxygen species pathways (28). Despite that SFN was reported to be a potent inducer of the Nrf2 gene (15, 42), no evident changes in the expression of any previously defined Nrf2-target genes were noted following a 1-year treatment (28). Additionally, when the effect on HDAC activity was tested, no significant changes were reported (9).

In breast cancer, SFN resulted in a significant modification of AKR1C1 and NQO1 expression (30), which are known to be altered in the DCIS (43–45). Accumulating evidence has shown that NF-E2-related factor 2 (Nrf2) and its downstream genes (e.g., NQO1 and AKR1C) exert a dual action on cancer, with both tumor suppressive and oncogenic effects (46–48). Under normal physiologic conditions, transient activation of Nrf2 and its target genes were reported to play a chemo protective role against cancer progression via regulation of oxidative stress, redox homeostasis, and metabolic reprogramming to anabolic signaling pathways (46–48). However, overexpression of Nrf2 can stimulate survival and proliferation of both normal and cancer cells; thereby, sheltering cancer cells from oxidative stress and apoptosis, and promotes their resistance towards chemotherapy (46–48). Accordingly, the use of molecules that transiently activate the Nrf2 pathway has emerged as a promising approach to protect against cancer development (49). Contrarily, Nrf2 related genes were reported to be upregulated and overexpressed in different cancers where they promote carcinogenesis and chemo resistance (46, 50, 51). Therefore, inhibition of Nrf2 may play a favorable role in advanced cancers during chemotherapy (52). Indeed, SFN was reported to exert a hormetic effect, which means it has a biphasic or a concentration-dependent response, in which lower doses could promote a favorable effect, while higher doses may lead to a negative/toxic effect (53). Wang et al. conducted pharmacokinetics and pharmacodynamics *in vivo* investigations on SFN and concluded that its effects on Nrf2 expression is mainly transient and that the levels returned to basal within 24h hours in rat lymphocytes unlike other molecules which had sustained the Nrf2 expression (54).

It has been also reported that the R-sulforaphane isomer would be more potent than its S-isomer on carcinogen‐detoxifying enzyme systems in cancer (55). Therefore, careful selection of sulforaphane preparation and dosing is warranted.

As stated earlier in the results, SFN treatment in DCIS patients resulted in a significant upregulation of NQO1 and a downregulation of AKR1C1 expression (30). The upregulation of NQO1 is expected to be a chemo preventive action as a result of the hormetic sub-toxic dose of SFN, which is also consistent with reported SFN actions on normal and cancer cells in preclinical studies (56, 57). However, the downregulation of AKR1C1 was ambiguous because it is inconsistent with previous studies on SFN (56, 57). Previous preclinical studies reported that SFN upregulates AKR1C1 through its action on the Nrf2; thereby, exerts a chemo-preventive role against cancer (56, 57). Actually, reference (30) is a clinical trial that was registered on clinicaltrials.gov and its results were posted on the same portal without publication in any journal. It has been included in our review as a grey literature to avoid any sort of reporting bias, which is a recommendation by many highly reputable sources, like Cochrane (23). We have tried to reach out to the authors for confirmation, but did not receive a response. If SFN truly reduced the expression of AKR1C1, then the investigational dose might have not only been a chemo preventive dose, but also a treatment dose to reduce cancer progression. However, there are contradictory literature reports on AKR1C1 impact on cancer prognosis; some studies reported that its downregulation is associated with better cancer prognosis while others stated the opposite (58–63). Nevertheless, further clinical studies and investigations are warranted to confirm the reported findings. Given the hormetic effect of SFN and the dual effects of Nrf2 in cancer, the therapeutic dose of SFN should carefully be selected and determined in future research for chemoprevention/anticancer uses. Furthermore, the role of Nrf2 and its target genes should further be explored in cancers to determine the role of various SNPs that could impact the variable reported expression patterns in normal versus cancerous cells.

Contrary to PCa, SFN led to a significant change in PBMC HDAC activity in breast cancer (9). When stratified by NSAIDs use, this change was principally in non-NSAID users (9). This may implicate a potential drug interaction between SFN and NSAIDs; therefore, future studies should further address and confirm the possibility of this interaction and its clinical significance. Of note, this was the only study reporting baseline use of other classes of medications (**Table S9**). On the other hand, no statistical significance was noted for Ki-67, H3K9ac, H3K18ac, HDAC3, HDAC6, and p21 levels in benign, DCIS, or IDC tissues following SFN intervention, which might be explained by the short duration of intervention (9).

In melanoma, there was no significant correlation between the administration of three different concentrations of oral SFN (50, 100, and 200 μmol) once daily for 28 days and changes in proinflammatory cytokines (29). Nonetheless, when the data were pooled from all dosage groups, statistically significant reductions in some cytokines were noted (29). The nonsignificant results might be due to the small sample size and short duration of follow-up. Therefore, further research should confirm these findings.

None of the studies reported significant adverse effects, which may support the safety profile of SFN and its feasibility as a potential future chemotherapeutic molecule. However, this should be further confirmed in larger well-designed clinical trials.

The present systematic review has some strengths that are worth mentioning. First of all, we implemented a rigorous and comprehensive search strategy following Cochrane Guidelines for Systematic Reviews of Interventions (23, 24). By default, this would mean that it has utilized the advantages of a well-conducted systematic review over literature reviews. That is, unlike literature reviews which are conducted to provide a thorough summary of the literature and deduce the research gaps, this systematic review provides support for evidence-based practice by summarizing and appraising the quality of existing specific types of studies. In addition, it was done according to a peer-reviewed priori protocol with rigid inclusion and exclusion criteria for certain study types and objectives, with no influence of the reviewer’s theory or belief regarding the topic (PROSPERO protocol ID: *CRD42022323788*). Besides, unlike the regular literature reviews, it utilized a rigid search strategy with specific domains and keywords corresponding to the prespecified inclusion/exclusion criteria. One more major advantage of this review is that we conducted a thorough search across multiple electronic databases, gray literature sources, and reference lists of relevant articles. This extensive search ensured that we minimized the risk of publication bias and included a wide range of studies, enhancing the generalizability of our findings. Therefore, it is a reliable up-to-date, and state-of-the-art systematic review that would support clinical decision making.

Despite the strengths mentioned, there are several limitations that should be acknowledged in this systematic review. First, the quality of the included studies varied, which may introduce heterogeneity into our findings. Moreover, variable formulations and dosing regimens were used and the results were limited to four forms of cancer, which makes it difficult to recommend a specific effective dose. Additionally, the potential for publication bias cannot be completely ruled out. Despite our comprehensive search strategy, it is possible that some relevant studies were missed, particularly those published in languages other than English. Therefore, although this systematic review benefits from a robust search strategy and rigorous data extraction methods, it is not immune to limitations stemming from the quality and diversity of the included studies, as well as the potential for publication bias. Recognizing these limitations is essential for interpreting the findings and guiding future research in this area.

## 5 Conclusion and future perspectives

The current literature has demonstrated that SFN is a favorable effective and safe therapeutic molecule for cancer treatment and chemoprevention. However, most of the available trials had high risks of bias, heterogeneity, and methodological weaknesses. Therefore, robust large-scale RCTs are warranted to further confirm the therapeutic potential of SFN, taking into consideration key oncological clinical endpoints. If further proven successful, new therapeutic molecules containing SFN need to be developed. We recommend to design and synthesis molecules that incorporate SFN chemical structure or its active functional groups based on a structure activity analysis. Additionally, to overcome the variable pharmacokinetic limitations of SFN administration, natural and synthetic compounds incorporating SFN moiety/active functional groups can be optimized and formulated into different drug delivery systems, such as liposomes, micelles, and nano emulsions and nanoparticles, which could result in a more targeted and selective delivery in pre-cancerous or cancerous tissues and better safety outcomes. Furthermore, variable dosage formulations and regimens were used in RCTs; therefore, the optimal formulation, dose, frequency, and duration need to be confirmed. The current data support SFN consumption chiefly in the early stages of cancers. Given the current suggested benefits, further investigations on severe cancer stages should be conducted. The current studies have also indicated favorable and excellent safety profile; however, highly powered studies should further confirm the short-and long-term safety of SFN.

## Data availability statement

The original contributions presented in the study are included in the article/**Supplementary Material**. Further inquiries can be directed to the corresponding author.

## Author contributions

DE: screening, data extraction, risk of bias assessment, writing initial draft, and design of illustrative figures. NA: screening, data extraction, risk of bias assessment, and reviewing and editing. AA: reviewing and editing. FA: reviewing and editing. AK: supervision, reviewing, editing, submission, and coordination among the team. All authors participated in the conceptualization of the research idea and protocol development. All authors contributed to the article and approved the submitted version.
