# Supplementary material for: Efficacy and tolerability of sulforaphane in the therapeutic management of cancers: a systematic review of randomized controlled trials
Source: Front Oncol. 2023 Nov 24;13:1251895. doi: 10.3389/fonc.2023.1251895 (PMC10710291; doi:10.3389/fonc.2023.1251895)
Supplement: Supplementary file 1 [file DataSheet_1.pdf]

Main databases search strategy

Table S1: Pubmed search strategy

**Database**	Pubmed
**Search strategy**	(Sulforaphane OR sfn OR broccoli OR 4-methylsulfinylbutyl isothiocyanate) AND ((cancer) OR (neoplasm) OR (carcinoma) OR (tumor))
**Filters**	Full text, Clinical Trial, Meta-Analysis, Randomized Controlled Trial, Systematic Review, Humans.
**Link**	https://pubmed-ncbi-nlm-nih-gov.qulib.idm.oclc.org/?term=%28Sulforaphane+OR+sfn+OR+broccoli+OR+4-methylsulfinylbutyl+isothiocyanate%29+AND+%28%28cancer%29+OR+%28neoplasm%29+OR+%28carcinoma%29+OR+%28tumor%29%29&filter=simsearch3.fft&filter=pubt.clinicaltrial&filter=pubt.meta-analysis&filter=pubt.randomizedcontrolledtrial&filter=pubt.systematicreview&filter=hum_ani.humans
**# of results**	**116**

Table S2: Embase search strategy

**Database**	Embase
**Search strategy**	('sulforaphane'/exp OR sulforaphane OR sfn OR 'broccoli'/exp OR broccoli OR '4-methylsulfinylbutyl isothiocyanate'/exp OR '4-methylsulfinylbutyl isothiocyanate') AND ('cancer'/exp OR cancer OR 'neoplasm'/exp OR neoplasm OR 'carcinoma'/exp OR carcinoma OR 'tumor'/exp OR tumor)
**Filters**	#1 AND ('clinical study'/de OR 'clinical trial'/de OR 'comparative effectiveness'/de OR 'comparative study'/de OR 'controlled clinical trial'/de OR 'multicenter study'/de OR 'phase 1 clinical trial topic'/de OR 'phase 2 clinical trial'/de OR 'phase 2 clinical trial topic'/de OR 'phase 3 clinical trial topic'/de OR 'pilot study'/de OR 'randomized controlled trial'/de OR 'randomized controlled trial topic'/de) AND ('controlled study'/de OR 'human'/de)
**Link**	https://www-embase-com.qulib.idm.oclc.org/#advancedSearch/resultspage/history.3/page.1/25.items/orderby.date/source.
**# of results**	**486**

Table S3: Cochrane search strategy

**Database**	Cochrane
**Search strategy**	(Sulforaphane OR sfn OR broccoli OR "4-methylsulfinylbutyl isothiocyanate") AND ((cancer) OR (neoplasm) OR (carcinoma) OR (tumor))
**Filters**	-
**Link**	https://www-cochranelibrary-com.qulib.idm.oclc.org/advanced-search
**# of results**	**112**

Table S4: Web of Science search strategy

**Database**	Web of Science

**Search strategy**	(ti=(cancer* or tumor* or carcinoma)) AND AB=(sulforaphane or sfn or broccoli OR 4-methylsulfinylbutyl isothiocyanate) and Articles (Document Types) and English (Languages)
**Filters**	Articles, English language, Title/abstract
**Link**	https://www.webofscience.com/wos/woscc/summary/a353e65b-10ce-4e66-9153-156e4288b018-2c9e1490/relevance/1
**# of results**	**765**

Table S5: Google Scholar search strategy

**Database**	Google Scholar
**Search strategy**	allintitle: Cancer AND Sulforaphane
**Filters**	Title
**Link**	https://scholar.google.com/scholar?hl=en&as_sdt=0%2C5&q=allintitle%3A+Cancer+AND+Sulforaphane+&btnG=&oq=allintitle%3A+Cancer+AND+Sulforaphane
**# of results**	**353**

Table S5: Natural Medicines search strategy

**Database**	Natural Medicines
**Search strategy**	Comparative effectiveness tool > cancer > Sulforaphane
**Filters**	None
**Link**	https://naturalmedicines-therapeuticresearch-com.proxy.lib.umich.edu/databases/food,-herbs-supplements/professional.aspx?productid=1070#effectiveness
**# of results**	**2**<br><br>• Cipolla BG, Mandron E, Lefort JM, et al. Effect of sulforaphane in men with biochemical recurrence after radical prostatectomy. Cancer Prev Res (Phila). 2015;8(8):712-9<br><br>• Alumkal JJ, Slottke R, Schwartzman J, et al. A phase II study of sulforaphane-rich broccoli sprout extracts in men with recurrent prostate cancer. Invest New Drugs. 2015;33(2):480-9.

Grey literature search strategy

Table S6: Clincaltrials.gov search strategy

**Database**	Clincaltrials.gov
**Search strategy**	Search #1: condition: cancer, intervention: sulforaphane or 4-methylsulfinylbutyl isothiocyanate. Search #2: condition: cancer, intervention: broccoli.
**Filters**	Interventional studies (clinical trials)
**Link**	Search#1: https://clinicaltrials.gov/ct2/results?cond=cancer+&term=&type=Intr&rslt=&age_v=&gndr=&intr=Sulforaphane&titles=&outc=&spons=&lead=&id=&cntry=&state=&city=&dist=&locn=&rsub=&strd_s=&strd_e=&prcd_s=&prcd_e=&sfpd_s=&sfpd_e=&rfpd_s=&rfpd_e=&lupd_s=&lupd_e=&sort= <br>Search #2: https://clinicaltrials.gov/ct2/results?cond=Cancer&term=&type=Intr&rslt=&age_v=&gndr=&intr=broccoli+&titles=&outc=&spons=&lead=&id=&cntry=&state=&city=&dist=&locn=&rsub=&strd_s=&strd_e=&prcd_s=&prcd_e=&sfpd_s=&sfpd_e=&rfpd_s=&rfpd_e=&lupd_s=&lupd_e=&sort=
**# of results**	Search #1: **21**, Search#2: **27**

Table S7: ProQuest dissertations search strategy

**Database**	ProQuest dissertations
**Search strategy**	ab(cancer or tumor or carcinoma) AND ab(sulforaphane or sfn or broccoli OR 4-methylsulfinylbutyl isothiocyanate)
**Filters**	Thesis, dissertations, English language
**Link**	https://www.proquest.com/pqdtglobal/results/ABEB5BB16A443BDPQ/1?accountid=49936
**# of results**	**170**

Table S8: ICTRP search strategy

**Database**	ICTRP
**Search strategy**	Condition: cancer* or tumor* or carcinoma AND Intervention: sulforaphane or sfn or broccoli OR 4-methylsulfinylbutyl isothiocyanate)
**Filters**	Recruiting status: ALL
**Link**	https://trialsearch.who.int/AdvSearch.aspx
**# of results**	**18**

Table S9: Baseline charchterstics of patients in the included studies

Publication	Age (Interv.)	Age (control)	Sex (Interv.)	Sex (control)	Race (interv.)	Race (control)	Co-morbidities (interv.)	Co-morbidities (control)	Other treatments (interv.)	Other treatments (control)	BMI (Interv.)	BMI (Control)
Zhang Z., 2020	65.7 (5.4)	64.9 (5.0)	100%, M	100%, M	96% white	95.8% white	Smoking 16%; Alcohol 26%	Smoking 20.8%; Alcohol 25%	N/A	N/A	28.9 (7.6)	31.1 (6.4)
Traka M., 2019	66 ±6; 66±6	68±5	100%, M	100%, M	N/A	N/A	N/A	N/A	N/A	N/A	27.6 ± 3.4; 27.7 ± 2.2	26.7 ± 3.1
Tahata S., 2018	46, 51, 44	46, 51, 44	F (50%, 67%, and 100%)	F (50%, 67%, and 100%)	Caucasian	Caucasian	N/A	N/A	N/A	N/A	NA	NA
Visvanathan K., 2018	52	54	100%, F	100%, F	N/A	N/A	N/A	N/A	N/A	N/A	NA	NA
Lozanovski V., 2019	62	68	55%, F	64%, F	NA	NA	NA	NA	NA	NA	25	25
Cipolla B., 2015	68.8 ± 6.4	70.4 ± 6.8	100%, M	100%, M	na	na	na	na	Prostatectomy + EBRT [19 (50%)]; Prostatectomy + EBRT + hormone therapy [6 (15.8%)]	Prostatectomy + EBRT [20 (50%)]; Prostatectomy + EBRT + hormone therapy [4 (10%)]	27.8 ± 4.1	26.5 ± 2.7
Atwell L., 2016	53.52 (9.54)	55.30 (14.27)	100%, F	100%, F	White: 26 (96.3%), Non-white:1 (3.7%)	White: 24 (88.95), Non-white: 3 (11.1%)	na	na	NSAIDS: 20 (74.1%)	NSAIDS: 8 (29.6%)	26.69 (5.38)	27.95 (5.80)
Traka M., 2008	64.38 ±3.64	62.5 ±5.29	100%, M	100%, M	NA	NA	NA	NA	NA	NA	27.76 ±2.45	27.37 ±3.99
